# Supplementary material for: Identification of Candidate Gene-Based Markers for Girth Growth in Rubber Trees
Source: Plants (Basel). 2021 Jul 14;10(7):1440. doi: 10.3390/plants10071440 (PMC8309273; doi:10.3390/plants10071440)
Supplement: Supplementary file 1 [file plants-10-01440-s001.zip › plants-1287724-supplementary.pdf]

plants

MDPI

Supplementary Materials

Table S1. ESTs used for ILP marker development.

Gene	Reference	Accession
CesA2	*Eucalyptus grandis*	EU165712.1
CesA3	*Eucalyptus urophylla*	JX271000.1
COBL4	*Eucalyptus nitens*	FJ213604.1
CaS	*Eucalyptus grandis*	XM_010028507.1
4CL	*Eucalyptus camaldulensis*	GQ916947.1
CAD	*Eucalyptus globulus*	AF038561.1
	Eucalyptus camaldulensis	GQ916948.1
	Eucalyptus pilularis	AB591253.1
COMT1	*Eucalyptus camaldulensis*	GU324973.1
	Eucalyptus camaldulensis	GU109375.1
CCoAMT	*Eucalyptus camaldulensis*	GU109373.1
	Eucalyptus cladocalyx	KC160171.1
	Eucalyptus camaldulensis	GU109372.1
CCR	*Eucalyptus pilularis*	AB591261.1
	Eucalyptus globulus	AY656819.1
C4H1	*Eucalyptus urophylla*	JX270996.1
	Eucalyptus urophylla	JX270997.1
PAL	*Eucalyptus robusta*	AB698855.1
Peroxidase2	*Eucalyptus pilularis*	AB591332.1
LIM	*Eucalyptus globulus*	AB591290.1
	Eucalyptus pilularis	AB591287.1
MYB1	*Eucalyptus globulus*	AB591297.1
	Eucalyptus pyrocarpa	AB591296.1
	Eucalyptus pilularis	AB591295.1
	Eucalyptus pilularis	AB591294.1
	Eucalyptus pilularis	AB591298.1
BTF3	*Eucalyptus grandis*	XM_010038713.1
	Eucalyptus grandis	XM_010071198.1
HD-zip	*Eucalyptus grandis*	XM_010055362.1
NAC1	*Eucalyptus grandis*	XM_010062528.1
	Eucalyptus grandis	XM_010037879.1
	Eucalyptus grandis	XM_010033715.1
	Eucalyptus cladocalyx	KC160157.1
APL	*Eucalyptus grandis*	XM_010065794.1
KNAT	*Eucalyptus grandis*	XM_010049851.1
NtLIM1	*Eucalyptus cladocalyx*	KC160161.1
	Eucalyptus globulus	AB208710.1
MOR1	*Eucalyptus grandis*	XM_010054049.1
PIN1	*Eucalyptus grandis*	XM_010038649.1
RIC1	*Eucalyptus grandis*	XM_010040929
FRA2	*Eucalyptus grandis*	XM_010035598.1
COB	*Eucalyptus globulus*	JX904063.1
KORRIGAN	*Eucalyptus camaldulensis*	HQ864573.1

Table S2. Characteristics of selected 115 ILP markers.

	Marker	Na	He	Ho	PIC	Forward Primer	Reware Primer	Tm (°C)	Expected Size (bp)
1	CeaA001_1	4	0.48	0.96	0.40	atacactagccaggttggtc	gtactattcctgccgtatgc	54.20	253
2	CeaA003	3	0.48	0.98	0.40	cattggtgagtactgcagag	gcctggaaataacacaagag	54.20	375
3	CeaA004	5	0.64	0.39	0.32	acagaggttgtcttgcttca	tgatcagggagaagaagatg	55.90	150
4	CeaA006	5	0.49	0.59	0.46	gaaccataaatccatccaac	tttattgcctccactctcat	54.30	250
5	CeaA009	2	0.51	0.87	0.37	ctagagatcaccctggaatg	ggagcattggtaagtactgc	54.70	283
6	CeaA011	5	0.51	0.55	0.43	tgtgattagctgtggctatg	gcatcttgaaaccagtaagg	54.90	176
7	CeaA012_2	4	0.62	0.32	0.38	ctttgccacttcttgcata	gcaatgaagaaaagacttgc	55.00	373
8	CeaA016	5	0.35	0.39	0.58	taggcttgataggaggttca	agaatgatcgatatgccaac	55.00	292
9	CeaA017	4	0.64	0.31	0.33	ggtaagaactgctgatacacg	tgagggtaatgaacttccac	55.00	193
10	CeaA019	3	0.54	0.04	0.40	agggtagtcaactgcaagaa	cgtggatattggatcagttc	55.00	299
11	CeaA020	3	0.57	0.05	0.37	gaaccagatactggcaatgt	cagctgtttgccttcttact	55.00	409
12	CeaA021	5	0.56	0.40	0.41	catgcatcttgaatcctgta	gatatgaggacaaggcagac	54.60	429
13	CeaA025	5	0.68	0.34	0.30	tgtgattagctgtggctatg	gcatcttgaaaccagtaagg	54.90	176
14	CeaA026_3	4	0.37	0.32	0.58	ttcatcatgccagaggtaat	gcaatgaagaaaagacttgc	55.60	317
15	CeaA027_2	4	0.58	0.28	0.41	agaaaagacttgcaaaggtg	tctgcttgctgacagataaa	54.70	338
16	CeaA028	2	0.52	0.80	0.36	gacaatgcatcttgaaacct	gattggggaactgaggtaat	55.20	159
17	CeaA029	4	0.55	0.57	0.40	gaaccagatactggcaatgt	ccttcttactgggaagttcat	55.10	399
18	CeaA032	3	0.42	0.91	0.36	tggaagttcattaccctcac	accagagatcatccaggaat	55.00	440
19	CeaA033	4	0.62	0.39	0.41	agatggctctccttctttct	cttcctgtaaaccgtgagac	54.80	165
20	CeaA034_2	3	0.59	0.45	0.37	gaaccagatactggcaatgt	ctgtcacatccattcctctt	55.10	441
21	CeaA035	3	0.55	0.27	0.39	catgcatcttgaatcctgta	agctgtggatatgaggacaa	54.60	450
22	CeaA036	2	0.11	0.12	0.10	taggcttgataggaggttca	agaatgatcgatatgccaac	55.06	292
23	CeaA037	4	0.32	0.59	0.63	ttaccctcactgtcaaatcc	ccggaaataacatcagagat	55.00	463
24	CeaA040_1	3	0.45	1.00	0.44	agtgcatcttgaatcctgtc	ccaagttatcagctgtggtt	55.20	200
25	CeaA043	3	0.49	0.99	0.38	gtccatttatccgaactttg	aaagtccacccatcttttg	54.70	279
26	CeaA044	3	0.72	0.33	0.25	ataatcaaccgcaagtatcg	gatcggctgtcactaaggta	55.40	341
27	CeaA046_2	2	0.50	0.94	0.21	agtgcattttgaatcctgtc	ccaagttatcagctgtggtt	55.20	195
28	COBL054	4	0.42	0.43	0.49	agtgaggaatgttcgcttt	ggctacacggtgagtagagt	54.90	297
29	COBL056	3	0.50	0.87	0.39	ctggagaaaacaaggatttg	tcaagcactgagtgagtttg	54.90	278
30	COBL057	7	0.23	0.54	0.76	aatgggatgtaatgtcttgg	ggtacatttggaagttgctc	54.80	390
31	COBL058	3	0.63	0.32	0.32	gaatttcactttgcttggtc	ggccagaaactgtgagtaag	54.90	241
32	COBL062	3	0.49	0.02	0.39	tgagtgttggtcaatctgg	taagtgcacgtcacattcc	55.30	399
33	COBL064_1	6	0.36	0.23	0.58	gctttgattacaagccactc	gcttccattaggacatcatt	55.10	261
34	CaS093	4	0.37	0.99	0.54	cacttttgttgagcatcgta	gtgtaagccccaaacataag	55.00	432
35	CaS094	4	0.60	0.46	0.37	gtttggggcttacactacag	gttgatgtctctcctccaga	54.90	222
36	CaS096	3	0.46	0.98	0.44	gtttgtccaggaatctcaag	tagaaagcaagtccatctcc	54.70	223
37	CaS105	8	0.21	0.66	0.77	atacttgaactgggattgct	gaagaagactgaacacaactga	53.90	329
38	CaS106	10	0.18	0.72	0.80	gcaacttcagttgtgttcag	gacggactacaaagcctcta	54.30	272
39	CaS108	3	0.51	0.83	0.35	ggctctttgctccttacata	attcccagctatcatctcct	54.80	295
40	CaS109	2	0.50	0.99	0.09	aggagatgatagctgggaat	gacctctcaatgtttggatg	55.30	468
41	4CL065	5	0.37	0.34	0.56	cttaagatcttgcctgatgg	agacaagtcccagatcactg	55.10	272
42	4CL066	5	0.42	0.36	0.54	agtgatctgggacttgtctg	ggtatgtgcaaaactcccta	55.10	499
43	4CL069	5	0.23	0.74	0.73	ctagcacatcctgacatatcc	cctttggaatggattctatg	54.80	385
44	CAD071_2	4	0.42	0.77	0.53	tccctgtgattgattttctc	cattgcaatttctcactcct	55.10	377
45	CAD072	6	0.32	0.69	0.63	acatcccctctggtattttt	agttgcaacccatgtaaatc	55.10	291
46	CAD073	4	0.35	0.61	0.60	ccgaacttagtcacctctga	gaagaatgatcttggcatgt	55.50	192
47	CAD074_2	2	0.50	1.00	0.28	atatcagtgtggcagattcc	ggtcctttctccttattcct	55.00	165
48	CAD075	3	0.75	0.27	0.24	ctgggatcctctctccttat	atatcagtgtggcagattcc	55.00	174
49	CAD076	5	0.36	1.00	0.57	ccatggttcctgggtatta	ccaacaataactccaactcc	55.70	200

50	CAD077	3	0.89	0.11	0.10	gtctacaccgatggaaaaac	ccatctggtattttcaccac	54.60	213
51	CAD078_2	5	0.61	0.35	0.40	tttctcactcctttggtcat	cagaattcaagcatctcctc	54.70	476
52	COMT079_1	6	0.28	0.77	0.67	tttctcactcctttggtcat	cagaattcaagcatctcctc	55.10	195
53	COMT081	4	0.39	0.77	0.54	ctccatccctttctggtatt	acgtaccttcatgaaaatgg	55.70	169
54	COMT082	3	0.59	0.51	0.34	tcctaaaggagatgccatt	gaagaatgaattcggctatg	54.80	289
55	COMT084	9	0.24	0.48	0.73	gccattttcatgaaggtaag	attttaagcagtgctcatcg	54.90	167
56	CCoAMT086_2	2	0.50	0.98	0.24	gaataccatggaaattggtg	ccagctttttcaattacagg	55.30	290
57	CCoAMT087_1	7	0.50	0.40	0.47	tgttgctcacaagattgact	tgtcagcatccacaaagata	54.20	195
58	CCoAMT088	3	0.46	0.99	0.33	tgtcagcatccacaaagata	ctgctcttccagttcttgat	55.10	185
59	CCoAMT090_2	5	0.37	0.89	0.52	agaagcatgctcaagaattg	gaattgacagcaaatcatcc	55.70	196
60	CCoAMT091	4	0.37	1.00	0.56	gctccccaggatatacacta	gagtctcttacagagtgatgctc	54.30	200
61	CCR110	4	0.33	0.97	0.57	atgaatcttgctggagtgac	cctttccatagcagtaccag	54.90	458
62	CCR111	3	0.50	0.35	0.38	atgaatcttgctggagtgac	cctttccatagcagtaccag	55.20	461
63	CCR112	3	0.49	0.83	0.36	atgaatcttgctggagtgac	ttcccatagcagtaccagtt	55.20	426
64	C4H113	6	0.31	0.45	0.65	gttgtgcttgaaagtttcct	aagatctgcaaggaagtgaa	54.60	283
65	PAL117	5	0.31	0.34	0.64	ttcccataggagaaccaag	tgctgtatgtggcaatatgt	55.10	759
66	Per170	2	0.92	0.09	0.08	gctctctttgaagaacatgg	gtagatgtgccctcgaaat	55.10	225
67	Per171	5	0.36	1.00	0.35	gtagatgtgacctcgaaatga	tctctttcaaggacttggtg	55.20	185
68	Per173	12	0.13	0.46	0.85	agtcttttcccctatgaagg	catctttgctcagattgcat	55.00	191
69	LIM118	5	0.33	0.54	0.63	gagttggatatggtgatgct	aggcaccagagaaaaatgt	55.00	266
70	LIM121_1	3	0.49	0.88	0.40	agagtaccccttcaaaggaa	atcactgcaaaggaacactc	55.50	474
71	MYB125	4	0.55	0.59	0.43	ctgttctgcctggtaatctc	aagacctgaccttaaacgtg	54.90	217
72	MYB126_1	5	0.38	0.86	0.58	tgttcttcctggtaatctcc	tcattatcaaactgcacagc	54.20	174
73	BTF129	3	0.78	0.12	0.23	cagtttgtaaatcccaaagg	acccctaacagcaaatacaa	54.80	238
74	HDzip134_2	4	0.33	0.88	0.61	tcctccaaggaagaagagat	ggtgaggacactctttgaac	55.50	165
75	HDzip135	5	0.42	1.00	0.29	gcaagtcaagttctggtttc	cgttctcttgcctgagtaat	55.00	191
76	HDzip137	6	0.40	0.85	0.44	gtttgcttctgttccctaga	agatagcacttggagctcag	54.70	271
77	HDzip143	3	0.80	0.18	0.18	ggcttaccaactgaagtgag	aaactggcatggtaatcatc	55.00	295
78	HDzip145	7	0.32	0.92	0.62	gaaaccagaacttgacttgc	agatatcaccgacacactcc	55.00	228
79	NAC146	4	0.86	0.09	0.15	gatctcaataagtgcgaacc	cggtttgttctcataccagt	54.90	249
80	NAC149	4	0.41	0.99	0.50	ctcctaaaggagagaaaacca	tcttgtggaaaaccctacac	55.40	349
81	APL155_2	4	0.48	0.51	0.52	ttaccctttaccacctcaaa	gttgctttccaagtctgaat	54.80	219
82	KNAT159_1	6	0.37	0.85	0.58	cacctggagaaactcctgt	tagagcagtccctgcaaa	55.00	199
83	KNAT161	4	0.35	0.66	0.59	caagtttggatggttcagat	tcctctctgatgtcagcaat	55.00	299
84	NtLIM165	4	0.49	0.99	0.06	gcttgagactttagcagcat	gtttggacaagagctttgag	55.00	282
85	NtLIM166_2	2	0.35	0.19	0.29	agagtaccccttcaaaggaa	atcactgcaaaggaacactc	55.50	474
86	NtLIM168	7	0.51	0.34	0.43	gatcaaatcttcaagcgaac	acggggtcaagtgaatataa	55.00	222
87	MOR199	4	0.36	0.54	0.59	ttcctaatttcaccatctcg	gtttgcggtctaaaaacaac	55.30	263
88	MOR202	3	0.49	0.89	0.37	caaaccacctcagaaggata	gcttgagatgctacagaagg	55.20	165
89	MOR205_1	2	0.55	0.68	0.37	caaaatgtctgacagaatgc	ggctactttaaccaccattg	54.20	177
90	MOR206	3	0.63	0.45	0.31	ccaatggtggttaaagtagc	ttggaagaggctaataaacg	54.90	274
91	MOR211	12	0.10	0.73	0.88	cctttctttctgaccacttg	actcctttggagaaatctgg	55.00	197
92	MOR213	4	0.46	0.94	0.43	gccacaatagcatcacaaa	gatgcgctgatagcttactt	55.60	191
93	MOR214	4	0.72	0.24	0.28	atctgccacggtcttctta	ttggctagtttatgcgattc	55.80	268
94	MOR219	2	0.52	0.81	0.37	caagatttgggaatggaag	tgaaaggtgaccctttattg	55.60	287
95	PIN220	3	0.54	0.32	0.38	cgtttggagttctagtgctt	cttgtcatcacacttgttgg	54.80	429
96	PIN221_2	3	0.52	0.54	0.42	tcgagcttaattggtctagc	ataatggcaggcatttctac	55.00	211
97	PIN222	4	0.25	0.80	0.68	cctgccattatagcaaaatc	gcaaagccatgaacagac	55.00	440
98	PIN223_2	4	0.39	0.95	0.54	acaccctgatattctcagca	ctcttcacaaccccaataaa	55.70	239
99	PIN227_1	5	0.54	0.24	0.39	gctgaaatcttctctttcca	gctcatgaccagaaagaagt	54.70	271
100	PIN229	2	0.50	0.90	0.37	taagaatcaaacccagtgct	gcagttatggctgtttcttc	55.00	281
101	RIC234	3	0.92	0.08	0.08	ccttttgaaaggcctaagat	ctatatgggcaacatgcttt	55.30	191

102	FRA235	3	0.70	0.32	0.29	tccttgggacatagatgaag	gctccttacgactctcaaaa	55.10	222
103	COB177	3	0.49	0.99	0.03	acaagaaggacaaggagtga	tcaagcactgagtgagtttg	54.80	214
104	COB178	2	0.50	1.00	0.13	gaattttggaatcacgactg	aattgttgcgtctctttctc	55.60	185
105	COB181	2	0.51	0.85	0.37	ctgcctctttctaaccgtaa	attgtgaccactgcctaca	54.90	294
106	COB182	2	0.92	0.08	0.08	tgagtgttggtcaatctgg	taagtgcacgtcacattcc	55.30	399
107	COB183	2	0.53	0.77	0.36	acaatcactccttgtccaac	cacagcagtaatggggtatt	54.90	342
108	COB184	4	0.57	0.50	0.39	gttgttcagcatccaaatct	aattctgactgcacatttcc	55.20	334
109	COB185	8	0.23	0.42	0.74	gcatatgacccattagatcc	ggtacatttggaagttgctc	54.40	430
110	COB186_2	3	0.45	0.68	0.54	ccatcttcttcacacctgat	aggccagaaactgtgagtaa	55.00	177
111	COB188_2	4	0.55	0.33	0.44	atcccaatctcaacaatgtc	tcatgccatagaacatacca	54.80	242
112	KORR191	3	0.62	0.45	0.35	aaggacatttcacaaccaga	cgtggtttaactggatcaat	55.60	193
113	KORR192	2	0.50	0.98	0.17	aactgatgtcttgcgtgaat	aaaacccacaacatagctca	55.70	185
114	KORR194	3	0.46	0.99	0.44	catactgaagtggctgaggt	acaaccagaccagcataatc	55.30	209
115	KORR196	4	0.47	1.00	0.30	tcacacaagaattccctctc	tccgtcggataattacactc	55.20	211
	Mean	4.20	0.47	0.59	0.44				
	Max	12	0.92	1.00	0.88				
	Min	2	0.10	0.02	0.06				

Table S3. Linkage Disequilibrium of 59 pairs based on estimated $r^2 \geq 0.1$.

LocusName1	LocusName2	r^2	Type of LD
LIM166_2	LIM121_2	0.64	Intragenic
CeaA036	CeaA031	0.57	Intragenic
LIM121_2	LIM121_3	0.51	Intragenic
LIM166_2	LIM121_3	0.46	Intragenic
APL155_1	APL153	0.43	Intragenic
MYB126_2	MYB125	0.31	Intragenic
CeaA006	CeaA002	0.3	Intragenic
HDzip145	HDzip143	0.21	Intragenic
KORR192	KORR191	0.18	Intragenic
CeaA036	CeaA026_1	0.15	Intragenic
HDzip145	HDzip134_2	0.15	Intragenic
CAD077	CAD075	0.15	Intragenic
PIN227_1	PIN220	0.13	Intragenic
COBL059_1	COBL059_2	0.13	Intragenic
HDzip134_1	HDzip134_2	0.11	Intragenic
MOR205_1	CeaA026_1	0.24	Intergenic
MYB126_2	4CL066	0.22	Intergenic
PIN227_1	MYB126_2	0.22	Intergenic
MYB126_1	MYB126_2	0.2	Intergenic
MYB126_2	CCR112	0.2	Intergenic
MYB126_2	LIM121_3	0.19	Intergenic
PIN220	MYB126_2	0.19	Intergenic
KORR191	MYB126_2	0.18	Intergenic
MOR205_2	APL155_1	0.18	Intergenic
COB188_2	NtLIM168	0.18	Intergenic
FRA235	MYB126_2	0.18	Intergenic
MYB126_2	LIM121_2	0.17	Intergenic
PIN221_3	MYB126_2	0.17	Intergenic
MYB125	CeaA035	0.16	Intergenic
MYB126_2	CeaA035	0.15	Intergenic
FRA235	PIN227_1	0.15	Intergenic
MYB126_2	CeaA002	0.14	Intergenic

APL155_1	CAD077	0.14	Intergenic
NtLIM166_2	MYB126_2	0.14	Intergenic
COB184	MYB125	0.14	Intergenic
CAD079_2	CeaA001_2	0.13	Intergenic
BTF129	MYB126_2	0.13	Intergenic
MYB126_2	CAD081	0.13	Intergenic
MYB126_2	CAD082	0.13	Intergenic
COB186_2	CeaA026_1	0.13	Intergenic
COB184	CAD082	0.13	Intergenic
LIM122_2	CeaA026_1	0.12	Intergenic
NtLIM166_2	CeaA026_1	0.12	Intergenic
MYB126_2	C4H113	0.12	Intergenic
MOR205_2	APL153	0.12	Intergenic
PIN227_2	CAD082	0.12	Intergenic
COB178	CeaA016	0.12	Intergenic
CCoAMT086_2	CeaA034_1	0.12	Intergenic
COBL062	CeaA026_1	0.12	Intergenic
LIM121_3	CeaA026_1	0.12	Intergenic
LIM121_2	CeaA026_1	0.12	Intergenic
COB184	CeaA046_1	0.12	Intergenic
PIN229	MYB126_2	0.11	Intergenic
COB184	MYB126_2	0.11	Intergenic
COBL058	CeaA034_1	0.11	Intergenic
MOR219	CAD082	0.11	Intergenic
LIM121_3	CeaA016	0.11	Intergenic
COB188_1	CeaA001_1	0.11	Intergenic
MYB125	CeaA046_1	0.1	Intergenic

Table S4. Association analysis of 115 ILP markers.

	GLM				GLM + Q				MLM + Q + K			
Trait	***Locus***	***p value***	***R^2***	***q value***	***Locus***	***p value***	***R^2***	***q value***	***Locus***	***p value***	***R^2***	***q value***
GiD	COBL064_1	1.1×10^{-9}	0.32	2.20×10^{-7}	COBL064_1	3.3×10^{-8}	0.26	7.1×10^{-6}	COBL064_1	2.0×10^{-7}	0.14	4.67×10^{-5}
	PIN227_1	7.9×10^{-7}	0.22	5.40×10^{-5}	PIN227_1	2.0×10^{-6}	0.19	1.4×10^{-6}	PIN227_1	9.9×10^{-6}	0.1	7.69×10^{-4}
	PIN220	4.1×10^{-6}	0.18	1.77×10^{-4}	PIN220	4.6×10^{-5}	0.14	2.0×10^{-3}	PIN220	1.0×10^{-4}	0.08	4.80×10^{-3}
	CesA3033	2.1×10^{-4}	0.15	6.20×10^{-4}	CaS108	2.5×10^{-4}	0.1	7.6×10^{-3}	CaS108	9.2×10^{-4}	0.05	0.03
	CaS108	3.1×10^{-4}	0.11	8.20×10^{-4}	MYB126_1	1.2×10^{-3}	0.12	0.02				
	PIN229	4.5×10^{-4}	0.07	0.01	C4H113	1.3×10^{-3}	0.19	0.02				
	C4H113	4.9×10^{-4}	0.22	0.01								
	CCR112	1.0×10^{-3}	0.11	0.01								
	MYB126_1	1.2×10^{-3}	0.13	0.01								
	CAD077	1.8×10^{-3}	0.07	0.01								
	CCR110	2.6×10^{-3}	0.13	0.02								
	PIN221_2	4.3×10^{-3}	0.09	0.03								
	MOR206	4.8×10^{-3}	0.06	0.03								
GiW	COBL064_1	3.6×10^{-9}	0.31	3.7×10^{-7}	COBL064_1	7.69×10^{-8}	0.26	8.28×10^{-6}	COBL064_1	4.32×10^{-7}	0.15	4.99×10^{-5}
	PIN227_1	3.5×10^{-6}	0.2	1.7×10^{-4}	PIN227_1	1.06×10^{-5}	0.18	5.70×10^{-4}	PIN227_1	4.80×10^{-5}	0.1	2.77×10^{-3}
	PIN220	3.0×10^{-5}	0.16	1.0×10^{-4}	PIN220	2.23×10^{-4}	0.13	7.60×10^{-3}	PIN220	4.66×10^{-4}	0.07	0.018
	CesA3033	5.5×10^{-4}	0.14	0.01	CaS108	6.00×10^{-4}	0.09	0.016	CaS108	1.80×10^{-3}	0.05	0.046
	CaS108	6.5×10^{-4}	0.1	0.01	MYB126_1	1.00×10^{-3}	0.13	0.023				
	PIN229	9.8×10^{-4}	0.06	0.01	C4H113	2.30×10^{-3}	0.18	0.038				
	C4H113	1.0×10^{-3}	0.21	0.01								
	MYB126_1	1.3×10^{-3}	0.13	0.01								
	CCR112	2.8×10^{-3}	0.09	0.02								
	CAD077	3.3×10^{-3}	0.07	0.03								
GiC	CAD076	1.1×10^{-3}	0.12	0.01	CAD076	1.2×10^{-3}	0.11	0.023	CAD076	1.6×10^{-3}	0.09	0.046
	CAD078_2	3.7×10^{-3}	0.11	0.03								

Table S5. Primers used for the amplification of *COBL* gene.

Name	Sequence
PT065-F1-M13F	/5AmMC6/TGTAAAACGACGGCCAGTCCAGGCATCATCCGTAGAC
PT065-R1-M13R	/5AmMC6/GGAAACAGCTATGACCATGCCTTGTACTCCGTTTATTTCACAA
PT064-F1-M13F	/5AmMC6/TGTAAAACGACGGCCAGTGCTTCTGCTGTTGTCTTTGC
PT064-R1-M13R	/5AmMC6/GGAAACAGCTATGACCATGGTGATTAGGAATTGGGCATGAATC
PT064-F2-M13F	/5AmMC6/TGTAAAACGACGGCCAGTCAAAGGGTTGGAAGTTGTTGAC
PT064-R2-M13R	/5AmMC6/GGAAACAGCTATGACCATGAAGTTGTCTAGAGTGCAATGGAT

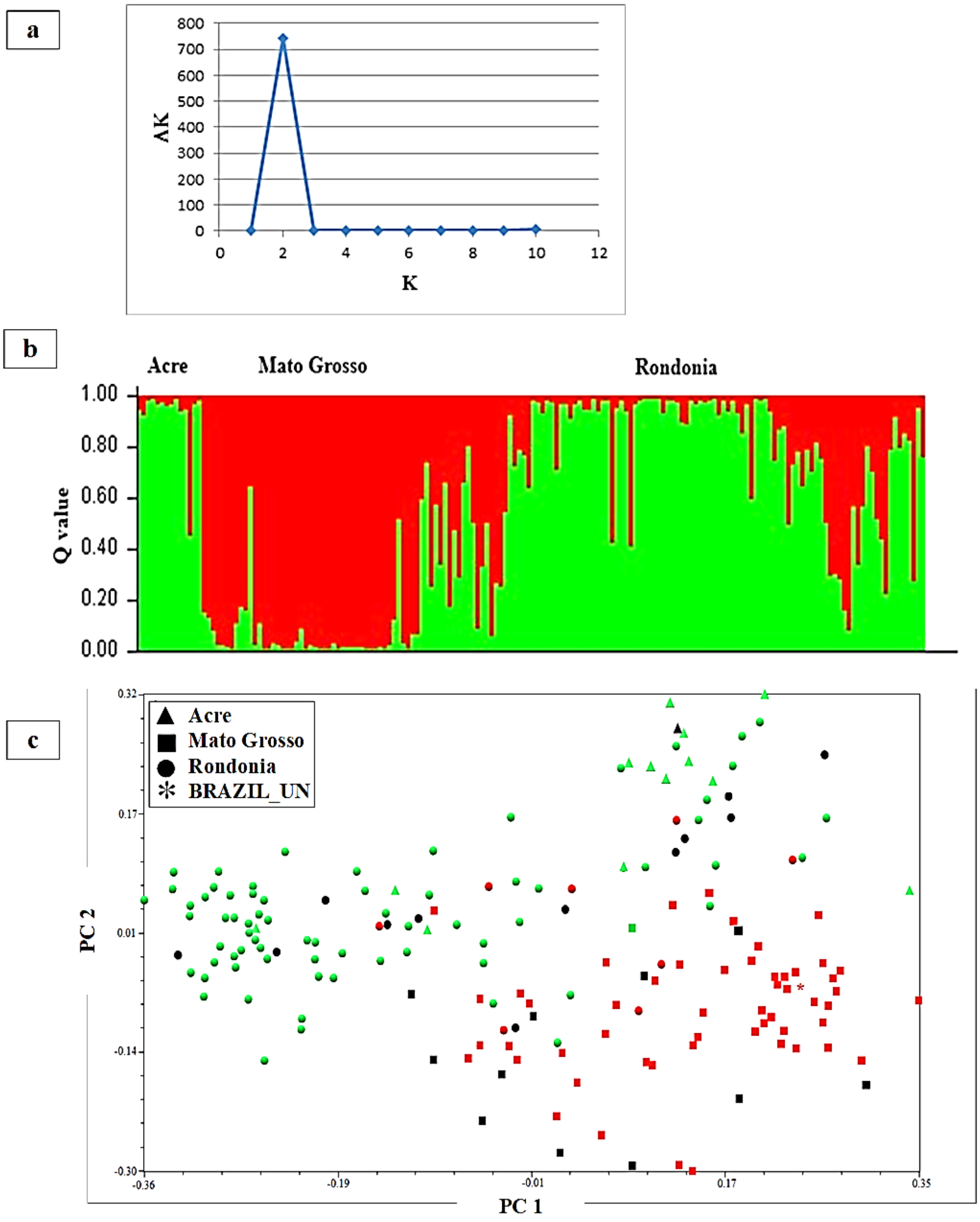

Figure S1. Population structure of 170 Amazonian accessions based on 115 ILP markers. (**A**) the most probable number of clusters (K), estimated using ad-hoc ΔK statistics (**B**) Bayesian analysis of population structure based on K = 2. Each accession is represented by a thin horizontal bar partitioned into two colored segments, whose length is proportional to the estimated membership coefficient (Q). (**C**) Principal component analysis (PCA). The triangles, squares, and circles, represent accessions from Acre, Mato Grosso, and, Rondonia, respectively.

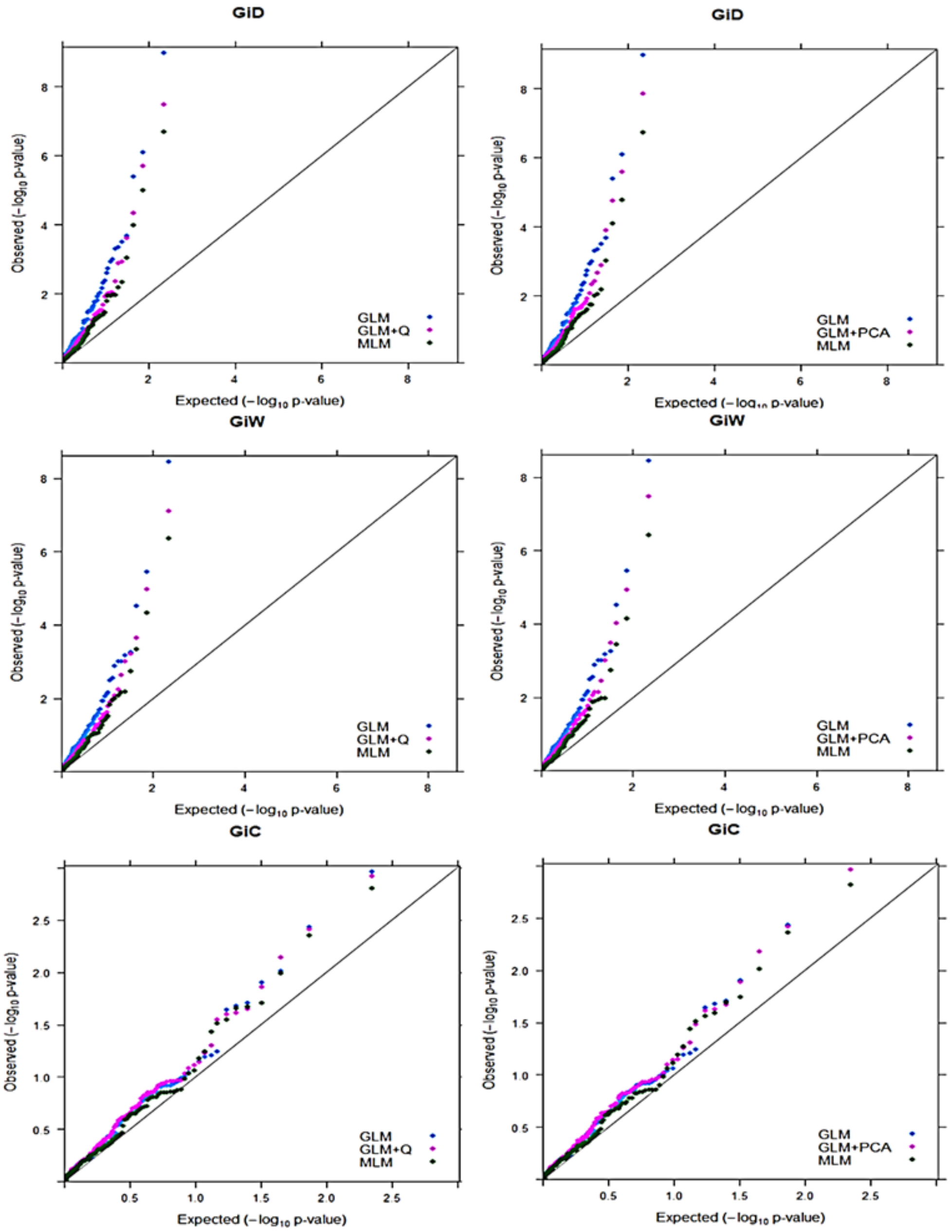

Figure S2. Quantile-quantile plots of estimated –log10 (*p*) from association analysis using three models and three traits: girth in the dry (GiD) and wet (GiW) seasons and total girth increment (GiC). The black line is the expected line under the null distribution. The blue line represents the observed *p* values using the GLM model, the red line those using the GLM with Q or PCA model, and the green line those using the MLM model with Q or PCA and K.
